# A credibility-driven evaluation of a community-based perinatal substance use disorder collaborative care model

**DOI:** 10.3389/fpubh.2025.1626095

**Published:** 2025-11-14

**Authors:** Christina M. Jäderholm, Teshanee T. Williams, Brad M. Wipfli, Lynne C. Messer, Liana B. Winett

**Affiliations:** 1 Department of Public Health, University of Copenhagen, Copenhagen, Denmark; 2 School of Public Health, Oregon Health & Science University – Portland State University, Portland, OR, United States; 3 School of Government, University of North Carolina, Chapel Hill, NC, United States

**Keywords:** perinatal care, substance use disorder, community-based, patient journey map, care coordination

## Abstract

**Introduction:**

Effectively treating substance use disorder (SUD) during pregnancy is critical to preventing adverse health outcomes for both parents and children, including overdose death and family separation. Although evidence supports investing in parental recovery through comprehensive care and support, these approaches remain under-examined, with community perspectives often marginalized due to evaluation challenges. This study evaluated the Substance Use Network (SUN) program, a community-based perinatal SUD recovery model in North Carolina.

**Methods:**

We used a patient-focused journey mapping approach to assess participant engagement, health outcomes, and alignment between participant and provider experiences. The evaluation used a mixed-methods approach, incorporating participant medical record review (*n* = 29), surveys (*n* = 29), focus groups (*n* = 7), and staff interviews (*n* = 11). Quantitative data assessed engagement metrics, treatment adherence, and birth outcomes. Thematic analysis of qualitative data from focus groups and survey responses provided insights into participants’ experiences with the program. Finally, interviews provided program staff perspectives.

**Findings:**

Participants were predominantly White non-Hispanic, all reported opioid use, most had polysubstance use, and 94% of participants maintained adherence to treatment. At the time of delivery, 87% of infants were born at term. Notably, 100% of infants born to parents enrolled in the first trimester were delivered at term. Through qualitative data, we identified areas of alignment and conflict between participants’ needs and organizational policies. Motivated by concerns for their baby’s health, participants emphasized opioid agonist treatment and non-judgmental, sustained support as key to recovery. Staff explained the importance of robust treatment and social service coordination, while recognizing a need for more training and sustainable funding.

**Conclusion:**

The journey map provides a comprehensive evaluation framework that enhances credibility and represents community perspectives meaningfully. This approach, which captures lived experiences alongside clinical outcomes, offers a replicable model for evaluating and strengthening community-based recovery programs. These insights can inform future improvements in perinatal SUD treatment and public health strategies to support pregnant and parenting individuals in recovery.

## 1 Introduction

Untreated substance use disorder (SUD) during pregnancy poses significant health and social risks, contributing to maternal mortality, with overdose being a leading cause of pregnancy-related deaths (1, 2). It is also associated with preterm birth and low birth weight (3–6). Postpartum SUD can disrupt parent–child bonding, increase the risk of infant injury and neglect, and lead to child welfare involvement and potentially family separation (7), all of which can negatively impact child development (8–12). In North Carolina, the percentage of out-of-home placements due to parental substance use increased from 36.5% in 2015 to 45.7% in 2022 (13). Yet, despite the growing prevalence of perinatal SUD in the state and beyond, fewer than 1% of pregnant women in the state receive specialized treatment (14).

Given the prevalence of perinatal substance use and limited access to treatment, family separation is a common consequence of parental SUD, codified as a child protective measure (15, 16). Breaking up families may not result in improved child outcomes and can cause additional harm. Children placed in foster care due to parental SUD face higher rates of depression, traumatic stress, and substance use disorders themselves compared to their peers who remain with their families who received SUD treatment and support (17).

Additionally, pregnant people with SUD experience high rates of housing insecurity and involvement with the criminal justice system (18). This can cause social isolation and estrangement from family and community support, which can further exacerbate health-harming social risks, increasing the likelihood of poor health outcomes (19). It is therefore crucial to address the social risks and barriers faced by pregnant individuals with untreated SUD to ensure the well-being of both the parent and their infant (20, 21).

Recovery health for pregnant and postpartum people with SUD depends not only on personal motivation, but also on systems of care, the policies that govern them, and social policy more broadly (22–25). The structural and social barriers to receiving SUD treatment include navigating complex and fragmented systems of care and fear of child welfare involvement, including potential loss of custody (26–28). Further, complex comorbidities and social instability can present barriers to navigating treatment services (29, 30), many of which do not accept pregnant patients (31).

Substance use is one of the most stigmatized health conditions (32), which has remained pervasive in the US, spanning policy (e.g., Medicaid reimbursement for substance use treatment), practice (e.g., substance use treatment protocols), and interpersonal environments (e.g., doctor-patient relationship and trust) (20, 33, 34). Perceived stigma and feelings of shame and guilt may prevent or delay pregnant people from disclosing their substance use (35); for many, fear of judgment and child welfare involvement is weighed against concerns for their baby’s health (36). Stigma can therefore deter pregnant individuals from seeking obstetric care and SUD treatment (37). Stigma can also lead to social isolation, making it difficult for women to disengage from drug use and establish ties to a non-drug-using world (19, 38).

The American Public Health Association and others have called for increased investment in evidence-based substance use treatment, emphasizing the need for comprehensive, multi-system approaches (39, 40). Further, the Substance Abuse and Mental Health Services Administration (SAMHSA) emphasizes that pregnant people with SUD require more than clinical interventions—they need compassionate, integrated support that includes mental health care, housing assistance, and childcare services (41).

Evidence-based interventions for perinatal SUD include medication-assisted treatment with opioid agonist therapies (OAT) such as buprenorphine and methadone (42, 43). Studies indicate that pregnant individuals receiving OAT have improved treatment retention compared to those who attempt abstinence-based recovery, and that parental abstinence or not having access to sufficient OAT can pose health risks, including return to use and overdose death (1, 42, 44). Psychosocial treatment is likewise important, but on its own has shown mixed results, reinforcing the need for comprehensive models that integrate medical and social services (45). Collaborative care models that bring together obstetric care, addiction treatment, behavioral health services, peer support, and social services have demonstrated success in improving maternal and infant health outcomes (46, 47). These programs facilitate increased engagement with prenatal care, reduce barriers to treatment, and promote long-term family stability (48). Despite their promise, such models remain under-examined, often due to funding constraints (49), which marginalize community perspectives on how to best treat SUD in pregnancy and postpartum.

To effectively develop and evaluate systems of care, enhance capacity, and replicate evidence-based models, it is essential to adopt a participant-focused framework. Such a framework must center the experiences and needs of those affected by SUD while also considering the strengths and limitations of the care system they navigate within. It is also important to develop a framework that considers the realities of community-based interventions, budgetary constraints, and the feasibility of sample sizes and data collection.

The Substance Use Network (SUN) in North Carolina was established in 2019 in Cabarrus and Rowan counties. SUN has taken a novel approach to comprehensive patient-centered care by building partnerships across the three sectors: health care, public health, and social services, including child welfare services to support pregnant women with SUD and keep families together whenever possible (see Appendix A for details). Key components include the SUN clinic, which offers SUD treatment and perinatal care, and partnerships with Atrium Cabarrus Hospital for childbirth education and care coordination. The initiative involves a multidisciplinary team trained in trauma-informed care and collaborates with organizations providing housing and family support.

The program is coordinated by the Suda Institute, a nonprofit that facilitates data sharing, financing, and cross-sector training to sustain the model. The Suda Institute’s role ensures alignment across sectors and supports program sustainability through coordinated funding and workforce development.

To enable coordination across sectors, SUN also addressed critical legal and regulatory barriers to information sharing. In partnership with UNC’s School of Government, the program developed a legal framework that complies with Federal and state confidentiality laws, such as 42 CFR Part 2, the Health Insurance Portability and Accountability Act, and child welfare regulations that needed to be addressed. SUN partnered with UNC’s School of Government to develop a legal framework that enables secure, cross-sector information sharing, making SUN one of the first programs of its kind in North Carolina.

To guide the evaluation of the SUN program, we used the Addiction Policy Forum’s *Patient Journey Map: Substance Use Disorder Treatment and Recovery* (50). This model, developed in collaboration with people with lived experience of SUD, outlines the phases of recovery. A “journey map” describes participants’ (e.g., patients or clients) feelings, experiences, and encounters as they move through the stages of their treatment or disease process. It thus illustrates the perspectives of those experiencing a health phenomenon, how they cognitively organize those experiences, and their emotional responses. Participants’ or patients’ maps of the lived experience of an issue can then inform more population-appropriate solutions.

While patient journey maps have also been used to analyze patient experiences across a continuum of care (51, 52)—most notably in cancer and mental health treatment—they have not yet been adapted for pregnant persons with SUD. In the present study, SUN program participants created a journey map that represents recovery phases as they experienced them. The map began with participants’ lives and experiences before entering SUN, and continued through birthing and transition into parenthood. This is important, as pregnancy is not an isolated event, but deeply connected to past experiences and circumstances, and affects health, sense of self, and social plight in the future. The patient journey map captured both individual and system-level facilitators and barriers to recovery, providing for an holistic approach to recovery treatment and supports (including OAT), prenatal care, resource coordination to meet social needs, and birthing care, all within a single community-based treatment model.

An often-overlooked aspect of program evaluation is credibility of the findings—the degree to which descriptions of situations, settings, and encounters resonate with those supplying the data (53, 54). Evaluating programs is critical to understanding individual’s lived experiences navigating complex systems. Thus, developing methods that credibly assess the realities of those impacted increases the relevance of the findings, helping programs more effectively respond to the needs of the populations they serve [e.g., (55, 56)].

Despite increasing recognition for integrated and supportive perinatal SUD treatment, significant gaps remain in research to evaluate them for responsiveness to participants’ needs (57). To address this gap, we examined the SUN program through two guiding lines of inquiry. First, we explored how pregnant people with SUD experience recovery within a community-based collaborative care model. Second, we investigated how a patient journey map framework can inform a credible evaluation of program effectiveness from both clinical and participant perspectives.

## 2 Methods

We used an interpretive phenomenological mixed-methods approach led by qualitative inquiry within a sample of 29 SUN participants from 2019 to 2023. Interpretive phenomenological analysis aims to describe a specific phenomenon, centering the experiences of the affected within the situational context of the physical and social environments in which the phenomenon takes place (58, 59). Our aim was to establish credibility in our findings while also offering a clear and replicable evaluation framework.

To evaluate the treatment engagement, health outcomes, and experiences of pregnant people working to recover from SUD within a perinatal treatment model, we structured data from multiple collection methods around a journey map. The primary focus of the journey map was participants’ experiences drawn from focus groups, surveys, and medical records. These findings were then supplemented by insights from program staff interviews to describe the broader care setting. The staff perspectives were used to identify the facilitators and constraints of providing care, offering contextual detail without shifting the focus away from the participants themselves. The study was approved by the Internal Review Board (IRB) at the University of North Carolina, Chapel Hill (Study #21-0326).

### 2.1 Research setting

The data for this study were collected by the SUN program (Table 1). Participants receiving care in the SUN clinic were recruited during two waves of data collection (April 15 – December 31, 2021, and September 15–December 31, 2023). SUN staff explained the study to participants in person or over the phone, and consent was given electronically. During both waves, data were collected through participant focus groups (*n* = 3 in 2021, *n* = 4 in 2023) and surveys (*n* = 16 in 2021, *n* = 16 in 2023). Additionally, participants’ records were reviewed for demographic information as well as health, social, and legal histories. There was no participation overlap between the two focus groups, but three focus group participants took the survey during both waves of data collection. While the actual count of participation invitations and acceptances was not collected for the interviews, staff reported that “almost everyone” agreed to participate when asked during the two waves of data collection. We therefore consider the sample representative of SUN’s participants at that time.

**Table 1:** Study overview: mixed-methods.

SUN participant data				SUN staff data
Method	Focus groups	Surveys	Medical records review	Interviews
Time and N	1st group: April, 2021 (*n* = 3) 2nd group: Sep. 2023 (*n* = 4)	1st wave: April-Dec, 2021 (*n* = 15) 2nd wave: Sep-Dec, 2023 (*n* = 14)	SUN participants enrolled any time from April 2019 to Oct. 2023 (*n* = 29, representing 34 pregnancies and 31 births*)	Sep. 2023 (*n* = 11)
Data	Qualitative data	Quantitative and qualitative data	Quantitative and qualitative data	Qualitative data
Foci	Participants’ experiences in the SUN program	Participants’ satisfaction and experience in the SUN program, motivation, and barriers to recovery	Participant demographics; medical, legal, and social history; diagnosis; social/medical needs; treatment history; birth outcomes	Staff’s experience working in the SUN program and perspectives on facilitators and barriers to recovery
Analysis	Thematic	Descriptive and thematic	Descriptive	Thematic
Integration	Motivators and challenges (individual and family) Medical and social histories (community)			Facilitators and barriers (systems)

*Some participants had two pregnancies while in the SUN program; one participant experienced a miscarriage, one left the program before giving birth, and one was still pregnant at the end of data collection.

Based on the interpretive phenomenological analysis approach, we analyzed the quantitative and qualitative data sources separately and then integrated the findings (59, 60). Traditionally, access to research participation and authentic representation in SUD research has been limited among women and pregnant people (61, 62). By collecting data through three modalities (focus groups, surveys, and medical records review), we offered different options for participant inclusion and expression to increase interest in participating in the study, validity of the findings, and credibility of the process (53, 63, 64).

### 2.2 Focus groups

The two focus groups were conducted in April 2021 (*n* = 3) and September 2023 (*n* = 4) and lasted approximately 50 min each. Focus group questions were standardized for consistency and prompted exploration of participants’ experiences in the program (see Appendix B). The focus groups were recorded and transcribed verbatim. No identifiers were collected; transcripts shielded names that were used in the conversations. Each participant was compensated $100 for their time.

We used thematic analysis (65) to iteratively organize participants’ narrative descriptions of events and phenomena into salient repeated ideas (codes) and further clustered these ideas into units of meaning (themes). The themes were guided by the phases of the conceptual framework (66). The transcripts were double-coded by two trained scientists [CJ; GK] who met to consolidate their codes. In cases of discordance, coders discussed differences in understanding and reviewed other examples until consensus was reached. Intercoder agreement was achieved at 89% across all variables. Analysis was done using MAXQDA software (2022 version) (67).

### 2.3 Surveys

Participant surveys assessed satisfaction with the SUN program. In 2021, the survey consisted of three 5-point Likert-scale questions with write-in options for comments assessing participants’ satisfaction with the program, perceived social needs, and access/barriers to care. In 2023, four questions were added, including two Likert-scale questions and two open-ended questions (write-in answers), together assessing participants’ motivation and barriers to staying in the SUN program (see Appendix B). For anonymity, no demographic information was collected. All survey respondents were compensated $50 for their time. On average, participants took 18 min to complete the surveys.

Survey responses with at least one question were retained for analysis, leading to the exclusion of three incomplete responses in 2021 and 2023. The final sample included 29 participants (*n* = 15 in 2021, *n* = 14 in 2023). We used descriptive statistics (counts and percentages) to summarize the Likert-scale questions. All are provided in Appendix B. The qualitative data from open-ended questions were coded using the thematic coding scheme developed from the focus group analysis (described below).

### 2.4 Medical records review

Participants’ medical records (*n* = 29) were reviewed in October 2023 for medical, social histories, engagement, and health outcomes. We also collected age, race/ethnicity, and county of residence. We retrieved numerical and categorical data as written (e.g., date of first visit, number of visits, diagnosis code). Data contained in record notes, such as social needs and past drug use, were converted into categorical variables. A full methodology and list of variables are provided in Appendix C.

From the medical records, we calculated three outcome variables. The first variable, *trimester of treatment initiation,* was calculated using gestational age at delivery, delivery date, and the date of the first visit to the SUN clinic and was categorized first (1–12 weeks), second (13–27 weeks), or third trimester (28–40 + weeks) (68). Dichotomous *preterm birth* was defined as birth before the 37th week of gestation (69), and *low birth weight* (defined as birth weight < 2,500 g) (70) was constructed among singleton live births. Additionally, we generated four categorical variables from the descriptive notes by identifying key terms; these variables included *substance use, trauma history*, *comorbidity*, and *social needs*.

The descriptive analysis provided numerical summaries (counts, percentages) across categories. Due to the small sample size and concern for anonymity, percentages are not reported for all outcomes.

### 2.5 Staff interviews

The interviews with SUN staff (*n* = 11) were conducted in September 2023 and lasted approximately 45 min each. Interview questions were prepared in collaboration with SUN’s program manager and standardized for consistency and prompted staff’s perspectives of the program (see Appendix D). The interviews were recorded and transcribed verbatim.

We conducted the interview analysis after analyzing the participants’ focus groups. We used thematic analysis to inductively and deductively organize staff’s descriptions into themes responsive to the journey map framework (65, 66). The transcripts were coded by CJ (who also coded focus groups) and discussed in depth with TW until consensus was affirmed. Analysis was done using MAXQDA software (2022 version).

### 2.6 Data integration

To integrate the findings from the focus groups, surveys, medical records, and interviews, each phase of the conceptual model was treated as a cluster of information. We read through the findings from each method and created an interpreted, cohesive description of participants’ experiences (60).

**Figure 1:**
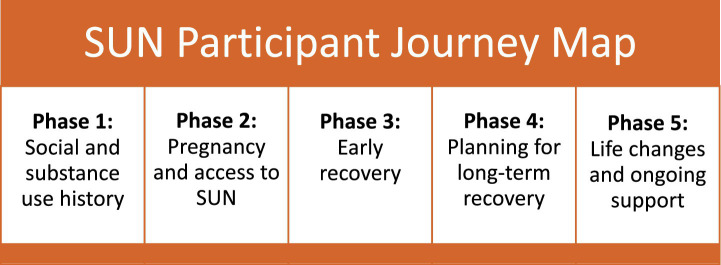
The SUN Participant journey map’s phases (1–5).

During the integration, the Addiction Policy Forum’s Patient Journey Map was adapted to better reflect the experiences of our sample. This included twice collapsing two distinct phases from the original journey map into one phase in our map. What constituted two salient phases for the population who developed the original framework were different for our (pregnant and postpartum) sample and became more meaningful as a single phase in their recovery journey. Our final conceptual model has five phases and is reflected in Figure 1. In each phase, we summarized qualitative findings and supported them with descriptive statistics. Last, the staff interviews provided programmatic context for each phase. Hence, the phases of the journey map integrate data from two or more sources (focus groups, surveys, medical record reviews, and/or interviews), depending on their relevance to that phase.

## 3 Findings

The final sample included 29 participants who experienced 34 pregnancies and 31 births (some participants had two pregnancies while in the SUN program, one participant experienced a miscarriage, one left the program before giving birth, and one was still pregnant at the end of data collection). The sample represented 37% of SUN’s 79 total participants from the 2019 program inception through the end of 2023.

### 3.1 Demographics

Among the SUN participants, 86% identified as White non-Hispanic, 14% identified as Black/African American. They were on average 28.3 years (range 20–36). Most participants lived within Cabarrus County (55%), while 27% came from the neighboring Rowan County, and 13% from other counties (Table 2).

**Table 2:** Participant demographics (*N* = 29).

Demographic characteristics	% (count)
Age at care initiation	
20–30	62% (18)
30–40	38% (11)
Race	
Black, non-Hispanic	14% (4)
White, non-Hispanic	86% (25)
County	
Cabarrus	55% (16)
Rowan	27% (8)
Other	13% (4)

Staff participants were from health care (clinic and hospital obstetrics, treatment, counseling, and peer support; *n* = 7), public health (care coordination, *n* = 1), and social services (Department of Human Services and non-profit housing/employment services, *n* = 3; Table 3).

**Table 3:** Staff participant descriptions and interview key.

Interviews (*n* = 11)		
SUN provider: obstetrics (*n* = 1)	Health Care	Interview #1
SUN provider: licensed clinical social workers (*n* = 2)		Interview #2
SUN staff: peer support specialists (*n* = 2)		Interview #3
Atrium hospital, delivery (*n* = 2)		Interview #4
Maternal care coordination (*n* = 1)	Public Health	Interview #5
Dept. human services (*n* = 2)	Social services	Interview #6
Endless opportunities housing services (*n* = 1)		Interview #7

### 3.2 Journey map

**Figure 2:**
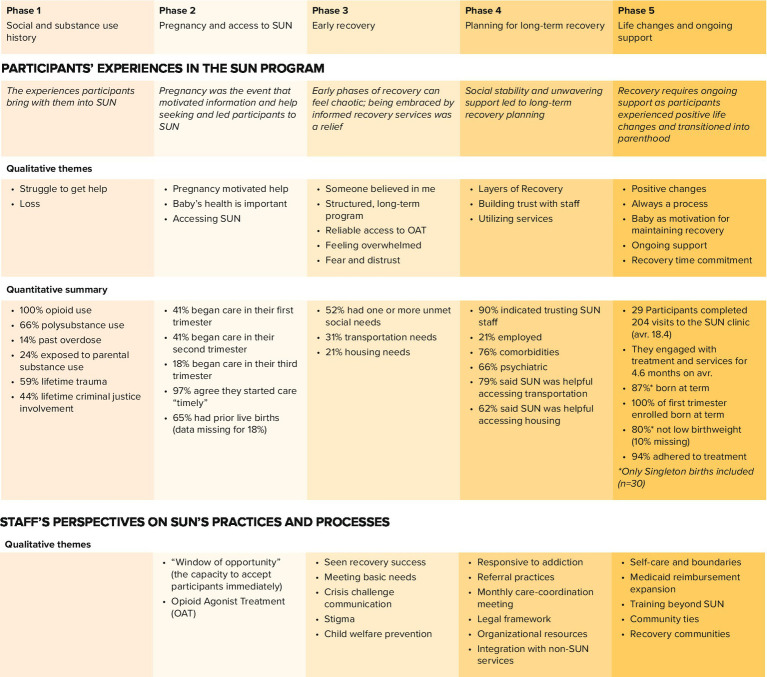
Study findings organized by the five phases in the SUN participant’s journey map.

The SUN patient journey map outlines the recovery stages identified by SUN participants (see Figure 2). Participants mapped their experiences prior to entering care and continued through their time in the SUN program. SUN program activities within these same phases were added by staff to further contextualize the participants’ experiences. The result is a framework (map) that evaluates SUN’s program from the view of its participants.

### 3.3 Journey map phase 1: social and substance use history

Phase 1 overview: participants’ health and social histories, and some prior experience with SUD treatment, constitute the lived experience participants bring with them into their SUN program-based recovery journey.

#### 3.3.1 Theme: struggle to get help

Participants summarized their lives with SUD as “a struggle to get help.” Many described past experiences encountering multiple barriers to entering -or staying in recovery programs. Barriers included long waitlists, not being eligible for treatment due to pregnancy, or not having a referral from a provider. One patient recounted her attempt to “be committed” to residential treatment:

*“I went to one hospital, and they said they had exhausted all the resources, because I came in willingly. So, I went to another hospital and lied and said that I was going to commit suicide. So, they committed me and eventually got me a place [to receive SUD treatment]” (2023).*

#### 3.3.2 Theme: loss

Another powerful reminder of what participants were up against in terms of addiction and barriers to receiving help was overdose death. Many had lost family members and friends to drug overdose and grieved the losses, but also the hopeless reality of SUD and dangerous drugs.

The medical records indicate SUN participants’ health and social histories. All participants reported past opioid use, ranging from prescription medications and diverted pain pills to non-prescription synthetic fentanyl and heroin. Two-thirds (66%) used multiple substances in addition to opioids, most commonly amphetamines and marijuana, reflecting the prevalence of polysubstance use among this population. For those who had a documented account of substance use initiation (*n* = 9), most began using in their teenage years, though some reported being introduced to opioids later in life through medical prescriptions. Further, more than half (59%) disclosed at least one traumatic experience, including physical abuse (31%), sexual abuse (21%), or other forms of trauma (27%). Nearly a quarter (24%) grew up in homes where alcohol or drugs were regularly used. Some noted being introduced to substances at an early age by family members. Many participants also faced involvement with the legal system: 44% reported a history of arrest, incarceration, or other legal challenges, and 14% had survived at least one overdose.

### 3.4 Journey map phase 2: pregnancy and access to SUN

Phase 2 overview: pregnancy was the event that motivated participants’ help and information seeking, which led to SUN engagement. Referral systems and the SUN’s approach set the parameters for treatment access and perinatal care.

#### 3.4.1 Theme: pregnancy motivated help

In focus groups, participants consistently indicated pregnancy as the event that prompted them to seek information and medical care, change their substance use behaviors, and/or enter treatment. Many had prior experience with sobriety or SUD treatment. Some expressed being “ready to quit” (2023) for a long time, while others reached a point of wanting treatment after learning about their pregnancy. Regardless of prior motivation, everyone saw pregnancy as a motivator and an opportunity to take steps towards treatment.

#### 3.4.2 Theme: baby’s health is important

To protect their own and their baby’s health, some participants had begun sourcing diverted Subutex (OAT) in their community. This means accessing prescription OAT without a prescription. Although pregnancy was not planned for many participants, all expressed concern for the health of their baby and wished they had *“a cheaper, easier route [to buy Subutex] than off of the street.” (2023)*.

#### 3.4.3 Theme: accessing SUN

Participants most commonly found the SUN program through a referral from a primary or urgent care provider after disclosing their substance use. A few participants had heard about SUN from community programs or by interacting with other SUD services. Participants indicated that access to SUN was fast and easy. They expressed relief to be able to begin care within days, as explained by a participant:

*“I was on fentanyl really bad. And I was I was actually coming in [Cabarrus County Health Alliance] with another girl, and I was going to the needle clinic […] the [receptionist] found out that I had just gotten pregnant, and she told me about it [the SUN program], and actually the same day — she probably dropped what she was doing -- got me into an ultrasound appointment. The same day. So, I mean, it was definitely a blessing, a blessing for that. It's definitely changed my life (2023).*

From medical records, initiation of prenatal care varied, with 41% beginning care during the first trimester and another 41% in the second trimester of pregnancy. The remaining 18% started in the third trimester. A majority (65%) had prior live births, with data missing for 18% of participants.

Survey responses indicate that 90% of participants “strongly agreed” they were able to begin care promptly. The remaining responses were split between “moderately agree” and “strongly disagree,” with a 3% non-response rate. These findings highlight the SUN program’s effectiveness in facilitating timely prenatal care access for pregnant individuals with substance use disorders.

#### 3.4.4 Staff descriptions

#### 3.4.5 Theme: “window of opportunity” (the capacity to accept participants immediately)

Staff described pregnancy as a crucial “window of opportunity” for initiating recovery. Pregnancy offers a time with heightened motivation and increased contact with health care and social services. The SUN program has the capacity to accept participants immediately, but is still working on a system to track referrals and time-to-initiation (time from first contact to first visit).

#### 3.4.6 Theme: opioid agonist treatment (OAT)

Staff are acutely aware of the potential negative health effects of substance use and the risk of return-to-use during pregnancy. Therefore, offering OAT is a crucial part of initiating treatment. Many participants experience polysubstance use, combining opioids with other substances such as cocaine or methamphetamine, for which there are no effective pharmaceutical treatments available. Based on staff experience, providing OAT can reduce the use of other substances, making it essential for facilitating recovery for pregnant individuals with complex substance use histories. To ensure that participants have immediate access to OAT at the correct dose, SUN collaborates with local pharmacies. Federal and state regulations govern the prescribing and dispensing of OATs like methadone and buprenorphine. These regulations can limit the availability of such treatments in certain areas, making collaboration with pharmacies essential to ensure patients have access to necessary medications.

### 3.5 Journey map phase 3: early recovery

Phase 3 overview: after accessing SUN’s program, the early phases of recovery can present a chaotic but also optimistic time. Several of the SUN program’s processes and practices are particularly central to participants’ recovery engagement during this time.

#### 3.5.1 Theme: someone believed in me

Participants agreed that a powerful and necessary facilitator for them to engage with recovery was having someone who immediately believed in them and communicated “*that they are wanted, that they deserve better” (2021)*. Some pointed to the peer support specialist – someone with lived experience – for giving them assurance and *“authentic advice”* (2023) during a difficult time. This steadfast support during the ups and downs of early recovery was explained as:

*“[The staff at SUN] care about you when you're at your lowest point. You know, feeling you don't deserve any better than that, like nobody cares, you have no hope, don't work towards anything, you don't care to do anything better, and that kind of stuff” (2021).*

#### 3.5.2 Theme: structured, long-term program

Participants also stressed the facilitating aspects of being in a structured, long-term program. They described the *“relief”* (2023) of knowing someone would follow them through pregnancy and the postpartum period.

#### 3.5.3 Theme: reliable access to OAT

Another key facilitator to staying engaged with recovery was consistently having reliable access to OAT. Participants described past experiences engaging in recovery and still having to source diverted OAT, opioids, or heroin to avoid withdrawal symptoms. During pregnancy, dosing needs change. Participants said they could trust SUN to help them with the prescription changes on short notice.

#### 3.5.4 Theme: feeling overwhelmed

Participants also described barriers to engagement, including feeling overwhelmed by the initial process at SUN and having to tell their story to multiple people. Some are alone in their recovery journey (without support from friends and family), and a few described being unprepared for the emotional burden of starting treatment. In surveys, 29% of respondents even indicated that “people in their life” limited their ability to access SUN services and support, with one respondent noting: *“not everyone in my life can see how the clinic helped me”* (2023). These barriers were also reflected in medical records, where a few participants indicated that a partner’s or family member’s active substance use conflicted with their recovery at SUN.

#### 3.5.5 Theme: fear and distrust

Another concern participants shared was fear of being reported to child welfare services. Many are wary of health care providers and described experiences of feeling judged, reprimanded, or taken advantage of as having created distrust. Fear is therefore quickly evoked by actions or attitudes from staff. A participant described how this fear manifested during one of SUN’s practices:

*“You wrote down everything that I would say, so that’s when I would start feeling like, is this some type of DSS [Department of Social Services] thing or [am I] being monitored or where is everything being written down going” (2021).*

In the survey write-in section, a few participants made note of not trusting some staff in the SUN program, feeling like staff’s thoughts or ideas were imposed on them, or they were judged for not being on time for their appointment. Further, one participant indicated that they strongly disagreed that they could trust the SUN staff.

### 3.6 Staff descriptions

#### 3.6.1 Theme: seen recovery success

The staff expressed a unanimous belief in SUN participants’ recovery success. They have observed what social support and stable OAT treatment can do to someone’s life, but also recognized that it can be a hard transition from street and prescription drugs to OAT. They emphasized the value of having peer support specialists to coach the transition to stable OAT use. The peer support specialists are able to *“be straight”* (staff interview) with participants because of their own lived experiences (see Tables 4, 5).

**Table 4:** Thematic findings from participant focus groups organized by the SUN participant journey map’s phases (1–5).

Phase	Theme	Description	*Quote*
Phase 1: Social and substance use history	Struggle to get help	A desire to get help and experiences with recovery treatment; Multiple barriers exist for entering into or staying in programs.	*“I was at [named place redacted], because they had to dispense your medicine, and you have to have a provider letter for them to give your meds. So, I got there. And there were like two days where I did not have my provider letter.” (2023)*
	Loss	The losses of friends and family due to drug overdoses affected participants and reminded them of the disease they were up against.	*“If there’s help and it’s available, then it should be given […] so we are not burying people before they have a chance to have a life and live up to their potential that they do have.” (2021)*
Phase 2: Pregnancy and access to SUN	Pregnancy motivated help	Pregnancy was the event that prompted participants to seek information, medical care, or enter into treatment	*“And I was about ready to quit when I found out I was pregnant. I went to [community resource center] where I live and they immediately suggested [the SUN clinic]. I called and got started”. (2023)*
	Baby’s health is important	Participants wanted to protect the health of their baby by decreasing their use or sourcing OAT without a prescription	*“I ended up coming [to SUN] to find the buprenorphine in a legal way and not buying it on the streets” (2023)*
	Accessing SUN	Access to the SUN clinic, including SUN’s practices, which facilitated engagement	*“I called and they call me right back. I got an appointment the next day” (2023)*
Phase 3: Early recovery	Someone believed in me	Having someone believe in participants’ recovery was a powerful and necessary facilitator to engage with recovery.	*[SUN…] make people know that they are wanted, that they do matter, that they should and that they deserve better […] addiction is a serious disease and a very hard, one very nasty one.” (2021).*
	Structured, long-term program	Knowing that SUN’s structural, medical, and social support services were available all throughout pregnancy removed an emotional burden from participants	*“And it was awesome to know that it was a long-term thing. [Pregnancy] was like, you are just gonna add the baby and be lost, and nowhere to go and no one to take care of you. That that long-term care is very helpful for an uncertain future.” (2023).*
	Reliable access to OAT	Having reliable access to OAT -correct dosing at the right intervals – was central to recovery engagement, particularly in the early phase of recovery.	*“I was put on medication to help just in case there was an [early] backslide and knowing that the support [for OAT] is there” (2021).*
	Feeling overwhelmed	Many participants were on their own when entering SUN. They felt overwhelmed by the initial process and having to tell their story to the staff.	*“And with pregnancy you do not really feel like being there all day and I was already tired after having to drive there and now it’s like I have to answer a thousand questions […] I just did not feel like being bothered and some stuff to me did not matter or did not pertain to what I was there for” (2021)*
	Fear and distrust	Past experiences led to distrust in medical and governmental systems; Fear of repercussions affected interactions with SUN staff	*“At first, I was skeptical of you, but that’s when I first met you, and it’s just because you asked so many questions, and there’s a reason for that.” (2023)*
Phase 4: Planning for long-term recovery	Layers of recovery	Progress can slow down with missed appointments and return to use; In recovery, it’s one issue at a time – one “layer” of challenge and response after another.	*“You know you make mistakes, or you know, [recovery] are two steps forward, one step backwards or two steps back, but you eventually get there” (2021)*
	Building trust with staff	Trust is a “two-way street”; Shared decision making around treatment is one key component. Participants are more honest about their progress when they receive genuine care and support for recovery.	*“The level of comfortability, the level of being heard and not just brushed off, being able to you know sit down and figure out a plan” (2021).*
	Utilizing services	Having access to resources and services became increasingly valuable as participants continued their recovery and planned for parenthood.	*“What’s helped me most from the services is being able to talk to [a licensed clinical social worker]. First and foremost, because I come from a very rocky past and being able to have confidence in somebody that you can count on is very comforting and reassuring” (2021).*
Phase 5: Life changes and ongoing support	Positive changes	Changes in habits, environments, and feeling empowered led to increased self-reliance and financial stability, resulting in new opportunities.	*“I have two other kids. And now I actually have one I’m getting back in custody because of this program.” (2023).*
	Always a process	Working through recovery is a constant process. It takes consistent work to not fall back on old reaction patterns when stressed or upset.	*“It’s a vicious cycle, so when you get accustomed to a lifestyle, you know that’s just all you know. Whenever you are stressed, mad or upset, or something goes wrong, then you revert back to that and it’s not the easiest thing to stay away from and to not go back to.” (2021).*
	Baby as motivation for maintaining recovery	Family needs, baby’s health, and being a good parent were motivators for maintaining recovery	*“I need to be the best mom I can be for [my children] so my sobriety is the most important. And the SUN clinic has helped me to be that mom” (2023).*
	Ongoing support	Resources and support systems are needed in the community to maintain long-term recovery and support a family	*“Housing for women and their children that are suffering from this, because it’s hard to do [alone]. I would love to see that.” (2023).*
	Recovery time commitment	Time is a barrier to continuous engagement; Time spent driving to and from appointments, time spent at the clinic, for many, now as a new mom	*“I do not live here so I have to sit in traffic […] I’m constantly busy. Like I have 5 kids. With the kids being in school and getting them situated. [Sometimes] it’d be a bit much overall when I come in” (2021).*

**Table 5:** Thematic findings from staff interviews organized by the SUN participants journey map’s phases (1–5).

Phase	Theme	Description	Quote
Phase 1: Social and substance use history			
Phase 2: Pregnancy and access to SUN	“Window of opportunity” (The capacity to accept participants immediately)	Pregnancy offers a time with heightened motivation and increased contact with health care and social services. SUN has the capacity to accept participants immediately when they feel ready.	*“There are plenty of patients out there who are just not coming in for care […] We do the best that we can and we are there” (Staff interview)*
	Opioid Agonist Treatment (OAT)	OAT is an evidence-based and necessary part of the treatment for opioid use disorder. Reliable access to this medication at the right dose facilitates engagement with the program and recovery success, even for participants with poly-substance use.	*“[Local pharmacies] working with us to help get those prior approvals and sending us a bill so [participants can] come in get their medicine […] it’s been that work over time of getting connected” (Staff interview)*
Phase 3: Early recovery	Seen recovery success	Staff believe in participants’ recovery. They have seen what stability and treatment can do.	*“It’s a rocky road, getting from heroin or prescriptive narcotics to buprenorphine […]Once through really, most of them do very well” (Staff interview)*
	Meeting basic needs	Meeting the social needs of participants (transportation, food, housing, social support, etc.) is seen as integral to recovery.	*“[recovery is easier for those who] already has the resources to sustain a life. Right. We have to give that to everybody. [People with] adequate social determinants of health do very well once you remove the drugs from their life” (Staff interview)*
	Crisis challenges communication	Participants often have instability, acute needs, or are in ‘crisis mode’, which challenges communication and places an emotional burden on staff.	*“Contact with our women is difficult sometimes because there’s not a lot of stability in their lives […] maybe their contact is not stable, or their housing is not stable. So, we might not even be able to do a home visit if we do not know where they are” (Staff interview)*
	Stigma	Stigma works to deter participants from engaging with treatment, prenatal care, and social services.	*“There are plenty of patients out there who are just not coming in for care […] The big things that keep them away is shame and stigma. We certainly do not stigmatize them when they come in. We praise them” (Staff interview)*
	Child welfare prevention	SUN’s goal is to preserve the parent–child dyad by preventing child welfare involvement; the Plan of Safe Care offers a preventative framework.	*“So, prevention [includes] do plan a safe care with them… we try to meet all the things that they are needing so that they can really focus on their recovery” (Staff interview)*
Phase 4: Planning for long-term recovery	Responsive to addiction	Set-backs, late or missed appointments, and/or return to use are expected. Sectors all align in supportive measures that encourage continuous treatment engagement.	*“there’s a lot of non-compliance. Unfortunately, not because they want to but because of other barriers that they face. [The care coordination manager] always make sure they have access to her, always available” (Staff interview)*
	Referral practices	Frequent communication between staff (a warm handoff) takes the burden off participants to navigate the system or re-tell their stories.	*“And with the nursing team too […] the client may already be saying, “I’ve got this going on in my life and it’s just wrecking me” and so that’s something that they do not have to necessarily repeat if the nurse can tell me “this is what’s going on” (Staff interview)*
	Monthly care coordination meeting	SUN’s partners meet monthly to coordinate care for participants; They discuss single cases and strengthen their internal practices.	*“[With] some of the patients […] there’s many people involved. [We] let them know upfront, we are here for you. We’re going to support you before they run away” (Staff interview)*
	Legal framework	SUN is built on a shared agenda and a legal framework that aligns goals and enables data sharing across agencies.	*“We can talk to the [DDS] caseworker and say, “Mom does not know what she needs to do […], what’s actually going on?” And they are a huge help” (Staff interview)*
	Organizational resources	SUN participants typically need extended resources and more time. Partners (organizations/agencies) must be aware of these long-term staffing and funding needs.	*“So, time and caseload. It takes more time to take care of women [with SUD] and what you even hear from them is “there’s a lot of people to see me”. And so, really, […] more time, means resources that actually pay for the service” (Staff interview)*
	Integrating with non-SUN services	Serving SUN-participants can be unpredictable and lead to “bottleneck” situations or staff being pulled in different directions. In organizations where SUN and non-SUN participants integrate, this can lead to staff frustration.	*“We hear things from [other staff] like, “Oh, they are the ones not coming on time,” or “they are the ones who are giving us a lot of calls. That is adding extra workload,” you know, but it’s like, well, that’s kind of part of their complicating circumstances” (Staff interview)*
Phase 5: Life changes and ongoing support	Self-care boundaries	Staff describe situations that can be emotionally taxing, particularly when responding frequently to participants in “crisis.” Monitoring workload, having self-care routines, and setting boundaries help them avoid burnout	*“There’s ability to have work-life balance, which is useful, again, to prevent burnout and make sure that this work can continue to be done […] I do not feel like I have to meet every single need” (Staff interview)*
	Medicaid reimbursement expansion	Reimbursement for services related to perinatal SUD treatment must match the time and resources that health and public health spend supporting recovery for pregnant people. Medicaid expansion is one way to fund programs.	*“Medicaid reimbursement rates are just not where they need to be to begin with. So, when [participants] come in and you spend an hour […] I mean, all the things that you have to do that takes time” (Staff interview)*
	Training beyond SUN	There is a need to train staff in SUD treatment at all partnering organizations. Training helps improve approaches but also counters SUD-related stigma.	*“We started with trauma informed care training, and we have repeated that multiple times. And so, we have a lot of internal training. We bring trainers in” (Staff interview)*
	Community ties	SUN’s sustainability is dependent on maintaining community ties through personal relationships within multiple sectors, such as health systems, correctional and legal systems, and with local policy and decision makers.	*“It started out of advocacy for our clients that would go to jail and not be able to get the appropriate treatment […] they still needed behavioral health support. We [continue planning this work] across the aisle so to speak” (Staff interview)*
	Recovery community	Building community resources to support long-term recovery goals, including housing for recovering families and colocation/expansion of services.	*“It’s difficult to maintain recovery when you do not know where you are going to lay your head down. Homelessness is a big trigger for a lot of women like if they are unstable, if they are on the street. They do not want to be on the street and be fully aware of that experience” (Staff interview)*

#### 3.6.2 Theme: meeting basic needs

Staff emphasize that addressing basic needs—such as transportation, food, housing, and social support—is critical during early recovery. A staff member explained that these needs provide the foundation for participants to remain engaged with OAT and benefit from services that foster long-term stability:

*“It can be hard to receive new information when in crisis or having a “full plate” just trying to survive. So we try to meet all [needs]… so that they can really focus on their recovery” (Staff interview).*

#### 3.6.3 Theme: crisis challenges communication

Staff may encounter challenges in communication and case management, particularly during the early stages of recovery. During that time, participants often face social instability, resulting in urgent needs and frequent crises. These situations demand rapid problem-solving and place both practical and emotional strain on staff, who must constantly shift between tasks. A staff member explains:

*“It can be a lot and mentally draining because we have clients who are living in crisis mode 24/7” (Staff interview).*

#### 3.6.4 Theme: stigma

Staff also see stigma as a consistent barrier to engaging with program-based recovery. They all have experience exhibiting or observing judgment and hostility in their field, and hear participants’ accounts of feeling ashamed and judged. To help counter the damage from stigmatizing experiences, staff emphasized the use of trauma-informed language in all encounters with participants. Still, the pervasive effects of societal stigma toward people with SUD can be difficult to overcome.

#### 3.6.5 Theme: child welfare prevention

Last, like participants, staff described *“fear of having baby taken away”* (staff interview) as a strong barrier to engaging with treatment. SUN’s social and clinical services are aligned in their goal of keeping families together whenever possible, which, among other practices, is formalized in their use of the federal provision, Plan of Safe Care (POSC). The POSC is a preventative tool aimed at safeguarding infant health by identifying substance-exposed pregnancies. SUN’s staff know that infant and parent health and well-being are intrinsically linked. By meeting basic needs and supporting mental, behavioral, and pregnancy health, they aim to avoid child welfare reporting and custody issues. Staff have seen these measures lead to positive outcomes. Yet, many times, participants’ prior experience with child welfare reports, investigations, and/or having children in out-of-home placement induces such stress that it continues to act as a barrier to engaging with prevention measures for their current pregnancy. Staff recognize the inherent conflict between recovery engagement and the threat of child welfare involvement. Ultimately, social service staff will transition from prevention services to child welfare services, as explained by staff:

*“Some people don't want us to come; the prevention side is a voluntary service. […] We are the car salesman, we're going to try and try until they're like “No, absolutely not.” But then also we have the child protective services side that's not voluntary” (Staff interview).*

### 3.7 Journey map phase 4: planning for long-term recovery

Phase 4 overview: Once past early recovery, plans for long-term recovery go through social stability, continued engagement with treatment, and utilization of social services to expand knowledge and skill building.

#### 3.7.1 Theme: layers of recovery

Participants expressed the value of working with providers who understand addiction and remain encouraging through the process. They talked about *“layers of recovery”* (2021), which includes missed appointments, times with slower progress, or return to drug use. Having providers who are non-judgmental and remain supportive is key to continuous treatment engagement, as described by one participant:

*“It was very easy to talk to anybody when you had a misstep here or there. They didn’t get mad at you or yell. It was just alright, it happened, now how do we get through this? They were awesome and kind. I mean being supportive and understanding. Even if they hadn't been through that walk of life themselves” (2023).*

#### 3.7.2 Theme: building trust with staff

When feeling seen and understood, participants said they could be honest about their recovery process and better engage with services and education. They explained that their trust in staff increased when staff were willing to share decision making power and were transparent about changes or challenges to treatment plans. A participant stated:

*“I feel like you're another number everywhere else. But [at SUN], you know, you're a person” (2023).*

#### 3.7.3 Theme: utilizing services

Access to services such as housing and transportation, plus referrals for mental health and family/parenting support, were described as core to the recovery process. By gaining stability and independence, participants felt empowered to plan longer-term and envision their future as a parent. Working through mental and behavioral health issues was described as a necessary part of the process, as one participant described:

*“I'm starting grief therapy, because I lost a son several years back. There's just so many things that I really didn't know that I needed help in to be able to stay clean” (2021).*

An examination of medical records and participant surveys from the SUN program provides insight into the socioeconomic and health challenges faced by participants and their experiences while receiving services. At the time of enrollment, 21% of participants were employed, with some actively seeking work. More than half (52%) reported one or more social needs, with transportation (31%) and housing (21%) being the most prevalent. The medical notes further highlighted that many participants faced unstable living situations, with housing needs evolving throughout their treatment. Changes included moving in with or separating from family members or partners, or seeking more space for their children. A significant majority (76%) had co-morbidities, with 66% experiencing psychiatric conditions such as depression, anxiety, and bipolar disorder.

Survey responses indicated that 90% of participants “strongly agreed” they were treated with respect within the SUN program. Regarding assistance with social needs: 59% found SUN to be “extremely helpful” in accessing housing resources, while smaller percentages reported it as “moderately helpful” (3%), “slightly helpful” (7%), or “not helpful/help not offered” (18%). In regard to facilitating transportation, 72% indicated that SUN was “extremely helpful,” with others rating it as “moderately” (7%) or “slightly” helpful (3%).

These findings underscore the complex interplay of social determinants and health challenges among SUN participants and highlight the program’s role in addressing these multifaceted needs.

### 3.8 Staff descriptions

#### 3.8.1 Theme: responsive to addiction

SUD affects all aspects of life, making recovery a process best supported by behavioral interventions, coaching, and skill building help. The staff expect that SUN participants will have setbacks, including late or missed appointments, return to use, and may engage in self-protective mechanisms through what a peer support specialist referred to as*“smoke and mirrors”* (staff interview). SUN is responsive to the realities of addiction and recovery through the treatment, services, and interventions.

As part of interventions, SUN offers behavioral health support and education to increase social and financial independence. Staff explained that as participants have their basic needs met, they also increase their capacity for receiving coaching and building skills. Each partner uses evidence-based tools and frameworks for intervention and education, and sees great value in having formal approaches. In the social service sector, they primarily utilize coaching techniques emphasizing practical skills such as budgeting and household management to help participants transition into long-term recovery and parenthood:

*“Budgeting is a big thing you know […] where they can cut corners and save. So really trying to say okay, let's lay out everything, all your income. And let's talk about the needs”(Staff interview).*

Within the health care and public health sectors, interventions include individualized behavioral health consultations or educational classes such as birthing, breastfeeding, infant care, and parenting. The curriculum is sometimes tailored to people with SUD, like legal aspects of pain management and giving birth while in recovery:

*“[“Prepare for childbirth” series] goes through pain management and has an attorney attached to it […] Because a lot of the questions that we get from moms are “How is my pain [going to be managed] and is there fentanyl in the epidural? And are they going to take my baby away as soon as I deliver?” (Staff interview).*

#### 3.8.2 Theme: referral practices

To ease transitions between treatment, services, and interventions, SUN has established a referral practice that includes both an electronic referral and direct communication between partners. The direct communication happens over the phone or in-person, known as a *“warm handoff” (Staff interview).* This practice removes the burden from participants to navigate the system and retell their stories.

#### 3.8.3 Theme: care coordination meeting

To further bolster referral practices and communication, SUN also has a monthly care-coordination meeting. Staff described the direct impact of getting all partners together to discuss complex care coordination cases for participants with extended needs. Having *“everyone in the room”* (*Staff interview*) can save time by not duplicating efforts or communicating in parallel.

#### 3.8.4 Theme: legal framework

To realize their referral practices and care-coordination meetings, SUN first had to establish a shared agenda and legal framework for exchanging participant information. Formalized and explicit alignment of goals has helped create trust between partners with different areas of expertise. Staff across agencies expressed a mutual understanding of treatment and support to aid participants’ recovery. The POSC, previously introduced in Phase 3, is an example of coordinating care across clinical and social services to anticipate and address risks before delivery in an effort to preserve families. This novel approach has, however, led to conflicts between participants’ recovery goals, monitoring, and reporting mandates. Still, SUN’s staff believed the blueprint they developed to improve collaboration across agency lines has increased SUN’s ability to effectively provide patient-centered care.

#### 3.8.5 Theme: organizational resources

Staff also identified organizational barriers that challenge their operations day to day, including constraints on organizational resources. As SUN continues to grow, each of the sectoral partners must plan for staffing and hours that will support the SUN program. Staff explained that it takes time and effort to treat and support people with SUD, and many staff are at capacity in terms of time and emotional availability. A SUN staff member in an administrative role explained:

*“We're super busy, and I'm only allowed to have one nurse [work] for me. That means I'm splitting my administrative time [to help out with SUN participants]… a huge barrier for me is trying to always budget and try to fight for additional hours” (Staff interview).*

#### 3.8.6 Theme: integration with non-SUN services

Another barrier occurs when SUN participants integrate with the (non-SUN) general population, receiving services through a partnering organization or agency (e.g., at the delivery hospital). SUN staff rely on nurses, medical assistants, and receptionists who serve the entire facility, not just the SUN program. Serving SUN participants can be unpredictable and lead to “bottleneck” situations or staff being pulled away from planned tasks. Among those staff not dedicated to SUN participants, this has led to agitation and frustration. As one health care staff member explained:

*“11 o'clock in the morning, and here [SUN participant] comes in, should have been there at 9.15. All of a sudden, all hell breaks loose because they [nursing staff] got other patients […] so it is very difficult […] and you just lose them, nurses that have stayed with us, which I didn't know was gonna happen” (Staff interview).*

### 3.9 Journey map phase 5: life changes and ongoing support

Phase 5 overview: Recovery is a process that changes beliefs and habits and can lead to positive life changes. This phase is conceptualized within the boundaries of active recovery at SUN and includes participants’ support needs, which are likely to continue beyond program-based recovery.

#### 3.9.1 Theme: positive changes

Participants described the gradual positive changes they experienced because of their recovery and enrollment in the SUN program. They talked about strengthening relationships with family members, regaining custody of children, securing housing, or taking steps towards becoming a peer support specialist. One participant shared how the SUN program helped her reconnect with her mother:

*“I’ve been able to open up and tell her more about things that I’ve done or seen when I was little [and when] I was addicted to drugs […]she's come to understand more from my point of view, not completely, but she's come around and that's all I can ask her—to try, you know” (2021).*

#### 3.9.2 Theme: always a process

Participants also expressed that recovery is a process and that changing habits takes time. Even when on a good path, they still must actively work towards their recovery goals.

#### 3.9.3 Theme: baby as motivation for maintaining recovery

Participants also consistently described that their family’s needs, baby’s health, or *“being a good parent”* (2021, 2023) were strong motivators for working to maintain recovery. As participants explained:

*“I have a little girl […] She is five months old. She was six pounds 14 ounces when she was born, she had no withdrawal symptoms, no signs of substance use, nothing like that. We both tested negative in the hospital. She's healthy she's growing well she's doing good, and without [SUN] I don't know that that could have been the outcome” (2021).*

*“I never thought I would get to where I am now, I never thought I’d be a mom, I never thought that I would do better” (2021).*

Participants agreed that the care they received through SUN’s program had set them on a path to long-term recovery. In surveys, most participants (86%) “strongly agreed” that the services and support they received at SUN would help them maintain long term recovery (7% “moderately agreed” and 7% were missing).

#### 3.9.4 Theme: ongoing support

Participants expressed the need for ongoing support. Some also talked about their need for community housing with access to residential or mobile OAT treatment. Some expressed a desire for a supportive or group housing situation for women with children. Last, participants suggested that SUN strengthen their approach by including a partnership with domestic violence resources.

#### 3.9.5 Theme: recovery time commitment

Maintaining recovery through SUN was also described as a substantial time commitment, which could present barriers to their continued engagement with treatment. Some wished they could access services closer to home as they transitioned into parenting and work. Time spent on transport and in appointments started to encroach on their life.

When summarizing participants’ engagement, birth-, and recovery outcomes from medical records, 29 participants had completed 204 visits to the SUN clinic (18.4 on average). They engaged with care and services for 4.6 months on average. Among singleton births (*n* = 30), 87% were born at term (>37 weeks completed gestation), and 80% of infants were not low birth weight (10% were low birthweight and 10% were missing birth weight data). At the time of birth, 94% of delivering participants adhered to treatment (defined as keeping appointments and filling MOUD prescriptions).

### 3.10 Staff description

#### 3.10.1 Theme: self-care and boundaries

When staff reflected on their work supporting SUN’s participants in recovery during pregnancy, they described finding meaning, but also a need for setting boundaries and practicing self-care. Situations in their work (e.g., responding frequently to participants in “crisis”) can be emotionally taxing. Peer support specialists particularly expressed not being able to continue their role long-term. Taking breaks and having time off and with family is very important, as a staff member notes:

*“If we were living in the emergency, in the crisis with our patients 24/7, that's a recipe for burnout. So, we can't do it” (Staff interview).*

#### 3.10.2 Theme: Medicaid reimbursement expansion

To financially sustain a program like SUN, where participants typically need extended services and time in appointments, leadership must either fight for local or state budget allocations or continuously prove their worth to secure grant money. A solution could be to extend Medicaid reimbursement for services related to clinical perinatal and SUD treatment, such as extended appointment time, time with peer support specialists, and breastfeeding support. Staff explain that “*sustainability [is only possible with] changing the funding model”* (Staff interview), as reimbursements do not currently match the time and resources it takes to support recovery for pregnant people.

#### 3.10.3 Theme: training beyond SUN

Funding is also needed for training staff across the sectoral partners collaborating with the SUN program, like the hospital’s emergency department and neonatal intensive care unit. Staff identify the value in training all staff interacting with SUN participants in managing care for patients with SUD. Trauma and recovery-informed approaches help prevent SUD-related stigma manifest in unsupportive language use, timeliness requirements, and hostile attitudes. Staff explain this could ease integration and improve care for people with SUD.

#### 3.10.4 Theme: community ties

SUN’s sustainability also depends on strong community ties. Founded in response to local needs, the program was built on professional relationships between healthcare, legal, correctional, and policy sectors. These relationships remain grounded in a shared understanding of the circumstances and social environment that determines health. To maintain community support, SUN staff actively educate decision-makers and local leadership about the realities of long-term recovery and recognize their roles as ongoing advocates and educators.

#### 3.10.5 Theme: recovery communities

To support sustained recovery, the Cabarrus County health department opened an additional clinic in 2021 where SUN participants can transition after one year postpartum. This clinic also provides services for participants’ romantic partners and family members in recovery. Despite this progress, housing remains one of the greatest obstacles for former SUN participants to maintain recovery. Staff envisioned expanding the postpartum phase of the SUN program into affordable housing ‘recovery communities’ that integrate services and formal family support.

## 4 Discussion

This study evaluated a community-based perinatal SUD recovery model using a patient-focused journey mapping approach. The findings capture the experiences of pregnant people navigating recovery within a collaborative care model. Findings demonstrate that recovery during pregnancy is a phased process shaped by personal motivation and social circumstances, system-level access, and structural supports—including trauma-informed care, OAT, and peer support. While participants overwhelmingly described the SUN program as life-changing, persistent barriers, such as the time commitment, fear of child welfare involvement, housing instability, and stigma, continued to constrain engagement and sustainability.

These results reinforce existing literature on the effectiveness of integrated perinatal SUD programs that align clinical, behavioral, and social supports. As others have described (48, 49), early and nonjudgmental access to OAT was key to recovery engagement. Participants in SUN echoed the importance of immediate, sustained care during pregnancy, an opportunity often lost due to restrictive policies, fragmented systems, and discriminatory practices (31, 71). Moreover, the central role of peer support reflects national calls for recovery-oriented systems of care that are culturally competent and inclusive of lived experience (41).

We also found that the SUN program struggles with sustainable funding. One specific area discussed by SUN’s staff is reimbursements from Medicaid, a joint Federal and State program to provide health insurance to eligible low-income individuals and families. Medicaid policy is governed by sitting administrations and can change in both concordance and conflict with health research demonstrating its positive effects on population health. As a sign of the times, Medicaid reimbursement schemes could decrease for care and treatment related to SUD. SUN’s evaluation findings are still relevant, but solutions may not be based on communicating scientific evidence demonstrating a need.

Public health aims to protect and promote community health while minimizing or eliminating health inequities. This study aligns those goals by integrating participant and provider experiences using a patient journey map. By grounding the evaluation in participants’ lived experiences and embedding their voices alongside clinical and organizational perspectives, the study challenged traditional top-down assessments that can marginalize the concerns of pregnant individuals with SUD. This approach revealed parallel tensions. For example, while both participants and providers identify pregnancy as a motivation for seeking treatment, participants also expressed fear that providers’ preventive intentions could lead to surveillance and custody loss. The dual realities of care and control, long documented in the literature on substance use and maternal health (27, 37, 72), were present in these tensions, reinforcing the importance of trust-building and transparency in collaborative programs.

### 4.1 Implications

**Figure 3:**
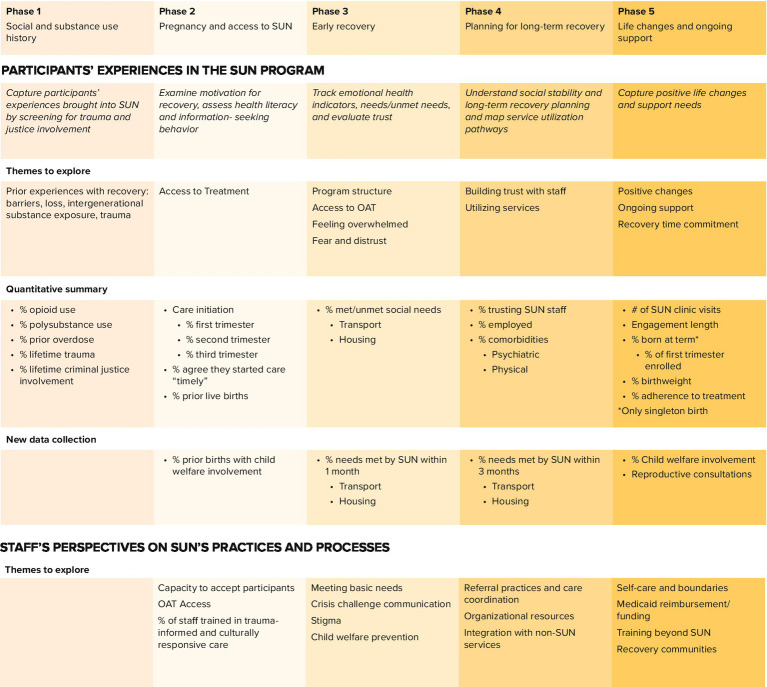
SUN’s ongoing evaluation framework centered around participants’ recovery journey.

The use of journey mapping as a participatory framework allowed participants to describe key recovery phases, reframing their experiences as ongoing, contextually grounded efforts toward healing. This study demonstrated how centering participant voices can strengthen trauma-informed and equity-centered evaluation practices that affirm the dignity and agency of people navigating recovery. By integrating these perspectives, this study offers a credible and resonant method for capturing outcomes that traditional evaluation tools may overlook, particularly for marginalized populations. This approach also produced a replicable framework for future evaluations, summarized in Figure 3, that can be implemented to monitor changes in engagement, retention, and participant well-being.

Building on these findings, we identified several actionable implications for the SUN program and similar community-based initiatives. Programmatically, expanding data collection to include meaningful, participant informed measures, such as time from referral to first visit, transportation and housing stability, access to reproductive health care, and both current and past child welfare involvement, will help track barriers to care and progress toward family preservation (73, 74). These metrics can be reassessed at multiple time points to monitor system responsiveness across recovery phases. At the policy level, the findings highlight the importance of extending Medicaid reimbursement to cover behavioral health, peer support, and perinatal recovery planning, while also investing in the infrastructure and workforce needed to sustain cross-sector collaboration (45, 75–79). Together, these actions provide a practical roadmap for strengthening community-based recovery systems and advancing family-centered, trauma-informed care for pregnant and parenting individuals with SUD.

### 4.2 Limitations

This study has limited generalizability due to a small sample size and its focus on a single program in one region. Self-reported data from focus groups and surveys may also introduce social desirability bias. Further, the focus groups were facilitated by a SUN member of staff, which could bias participants towards favorable perspectives. We prioritized participants’ comfort and trust and further mitigated these limitations by using multiple data sources. By combining focus groups, surveys, medical records, and staff interviews, the analysis provides a triangulated understanding of participants’ experiences (80). While the findings may not be universally applicable, they offer valuable insights into structuring and sustaining a collaborative, trauma-informed model where trust, flexibility, and participant agency are crucial for engagement.

## 5 Conclusion

Overall, this study demonstrates the value of journey mapping as a participatory approach for evaluating complex, community-based interventions for perinatal substance use disorder. By structuring the evaluation around participants’ lived experiences, journey mapping provided insight into the recovery phases, the relational dynamics between participants and providers, and the systemic boundaries that shape access to care. We found that stigma, fear of surveillance, and policy fragmentation presented barriers to recovery. We present a method that integrates clinical, public health, and social service perspectives while preserving the voices and agency of people navigating recovery within these systems.

Beyond documenting these findings, this study contributes a replicable framework for assessing *how* and *for whom* collaborative care models are effective. Journey mapping can guide future research by identifying participant-defined indicators of success, informing the design of longitudinal evaluations, and helping systems monitor change over time. As public health systems continue to grapple with rising maternal overdose and deep-rooted health inequities, journey mapping offers a compelling tool for designing, evaluating, and sustaining more responsive, equitable models of care.

## Data Availability

The raw de-identified data supporting the conclusions of this article will be made available by the authors, without undue reservations.
