# Supplementary material for: A credibility-driven evaluation of a community-based perinatal substance use disorder collaborative care model
Source: Front Public Health. 2025 Nov 14;13:1626095. doi: 10.3389/fpubh.2025.1626095 (PMC12660230; doi:10.3389/fpubh.2025.1626095)
Supplement: Supplementary file 1 [file Supplementary_file_1.pdf]

Appendix B: SUN participants survey results by year (2021 and 2023) and combined.

Table 4a: Q3 (n=29)<br>21: n=15<br>23: n=14	Strongly agree			Moderately Agree			Slightly Agree			Slightly Disagree			Strongly Disagree			Missing		
	21	23	Co	21	23	Co	21	23	Co	21	23	Co	21	23	Co	21	23	Co
I feel **safe** in the SUN Clinic	87% (13)	93% (13)	90% (26)	0%	7% (1)	3% (1)	0%	0%	0%	0%	0%	0%	7% (1)	0%	3% (1)	7% (1)	0%	3% (1)
I was able to begin treatment at the SUN Clinic in a **timely** manner.	87% (13)	93% (13)	90% (26)	0%	7% (1)	3% (1)	0%	0%	0%	0%	0%	0%	7% (1)	0%	3% (1)	7% (1)	0%	3% (1)
I feel like I am receiving the **help and support** I need from SUN Clinic providers	73% (11)	93% (13)	83% (24)	13% (2)	7% (1)	10% (3)	0%	0%	0%	0%	0%	0%	7% (1)	0%	3% (1)	7% (1)	0%	3% (1)
I feel that I can **trust the partners** of the SUN collaboration network	87% (13)	93% (13)	90% (26)	0%	0%	0%	0%	7% (1)	3% (1)	0%	0%	0%	7% (1)	0%	3% (1)	7% (1)	0%	3% (1)
I feel like the people who work here treat me with **respect**	87% (13)	93% (13)	90% (26)	0%	0%	0%	0%	7% (1)	3% (1)	0%	0%	0%	7% (1)	0%	3% (1)	7% (1)	0%	3% (1)
I am **satisfied** with the services that I have received at the SUN Clinic	80% (12)	93% (13)	86% (25)	7% (1)	7% (1)	7% (1)	0%	0%	0%	0%	0%	0%	7% (1)	0%	3% (1)	7% (1)	0%	3% (1)
I feel like SUN Clinic providers work together well with other agencies to provide me with the **support** that I need	87% (13)	93% (13)	90% (26)	0%	0%	0%	0%	0%	0%	0%	7% (1)	3% (1)	7% (1)	0%	3% (1)	7% (1)	0%	3% (1)

Table 4b: Q4 (n=29)<br>21: n=15<br>23: n=14	Extremely Helpful			Moderately Helpful			Slightly Helpful			Moderately Unhelpful			Not helpful/ Not offered			Missing		
	21	23	Co	21	23	Co	21	23	Co	21	23	Co	21	23	Co	21	23	Co
How helpful is the SUN Project with getting you access to transportation?	67% (10)	79% (11)	72% (21)	7% (1)	7% (1)	7% (2)	0%	7% (1)	3% (1)	0%	0%	0%	13% (2)	7% (1)	10% (3)	13% (2)	0%	7% (2)
How helpful is the SUN Project with getting you access to housing?	40% (6)	79% (11)	59% (17)	7% (1)	0%	3% (1)	7% (1)	7% (1)	7% (2)	0%	0%	0%	20% (3)	14% (2)	18% (5)	27% (4)	0%	14% (4)
How helpful is the SUN Project with getting you access to employment?	40% (6)	71% (10)	55% (16)	7% (1)	0%	3% (1)	7% (1)	7% (1)	7% (2)	7% (1)	0%	3% (1)	20% (3)	21% (3)	21% (6)	27% (4)	0%	14% (4)
How helpful is the SUN Project with getting you access to parenting support?	73% (11)	86% (12)	79% (23)	7% (1)	14% (2)	10% (3)	7% (1)	0%	3% (1)	7% (1)	0%	3% (1)	7% (1)	0%	3% (1)	0%	0%	0%
How helpful is the SUN Project with getting you access to child care assistance?	73% (11)	71% (10)	72% (21)	0%	0%	0%	7% (1)	7% (1)	7% (2)	0%	7% (1)	3% (1)	7% (1)	14% (2)	10% (3)	7% (1)	0%	3% (1)
How helpful is the SUN Project with getting you access to counselling?	93% (14)	93% (13)	93% (27)	7% (1)	0%	3% (1)	0%	0%	0%	0%	7% (1)	3% (1)	0%	0%	0%	0%	0%	0%
How helpful is the SUN with getting you access to relationship/family support?	87% (13)	86% (12)	86% (25)	0%	7% (1)	3% (1)	7% (1)	0%	3% (1)	7% (1)	7% (1)	7% (2)	0%	0%	0%	0%	0%	0%

Table 4c: Q5 (n=29)<br>21: n=15<br>23: n=14	Strongly agree			Moderately Agree			Slightly Agree			Slightly Disagree			Strongly Disagree			Missing		
	21	23	Co	21	23	Co	21	23	Co	21	23	Co	21	23	Co	21	23	Co
**Transportation** problems limit my ability to **access** the SUN Clinic in person.	27% (4)	21% (3)	24% (7)	7% (1)	0%	3% (1)	7% (1)	7% (1)	7% (2)	0%	7% (1)	3% (1)	47% (7)	43% (6)	45% (13)	13% (2)	21% (3)	17% (5)
**Virtual visits** allow me to access services I need at the SUN Clinic I otherwise may not have been able to access	67% (10)	50% (7)	59% (17)	7% (1)	0%	3% (1)	0%	14% (2)	7% (2)	0%	7% (1)	3% (1)	13% (2)	7% (1)	10% (3)	13% (2)	21% (3)	17% (5)
I feel the SUN Clinic has allowed my baby/children to be a part of the treatment and **recovery process**	60% (9)	71% (10)	66% (19)	0%	7% (1)	3% (1)	13% (2)	14% (2)	14% (4)	7% (1)	0%	3% (1)	7% (1)	0%	3% (1)	13% (2)	7% (1)	10% (3)
I feel that the services and support provided by SUN has helped me to be stronger or more **self-reliant**?	67% (10)	86% (12)	76% (22)	13% (2)	7% (1)	10% (3)	0%	0%	0%	0%	0%	0%	7% (1)	0%	3% (1)	13% (2)	7% (1)	10% (3)
***People in my life** limited my ability to access SUN services and support		29% (4)	29% (4)		7% (1)	7% (1)		0%	0%		0%	0%		57% (8)	57% (8)		0%	7% (1)
*I feel the services and support I receive at the SUN clinic will help my long-term **recovery goals**		86% (12)	86% (12)		7% (1)	7% (1)		0%	0%		0%	0%		0%	0%		0%	7% (1)

* Added in 2023

Appendix B: SUN participants survey results by year (2021 and 2023) and combined.
