# Supplementary material for: A credibility-driven evaluation of a community-based perinatal substance use disorder collaborative care model
Source: Front Public Health. 2025 Nov 14;13:1626095. doi: 10.3389/fpubh.2025.1626095 (PMC12660230; doi:10.3389/fpubh.2025.1626095)
Supplement: Supplementary file 2 [file Supplementary_file_2.pdf]

Appendix C: SUN participants records review methodology

Methodology: The records were reviewed once between Sep. 20-Oct. 6, 2023. The participants' demographics, diagnosis codes, dates of visits to the SUN clinic, and birth outcomes were collected from specific fields of the record, all other data was abstracted from note fields. For medical-, legal- and social history, as well as social needs, we used daily notes from the participant's first visit and first assessment. For some participants we also had access to care coordination assessments from Cabarrus County Health Alliance, including social needs assessments, social- and medical history, and birth outcomes. We cross referenced and supplemented missing data with care coordination notes when possible.

Table 1- participant demographics and history:

Diagnosis for Substance Use Disorder (SUD) and Opioid Use Disorder (OUD) were determined based ICD-10 diagnosis codes.

SUD included: F15.10 Other stimulant dependence, uncomplicated; F15.11 Other stimulant dependence, in remission; F18.20 Inhalant dependence, uncomplicated; F18.21 Inhalant dependence, in remission; F19.10 Other psychoactive substance abuse, uncomplicated; F19.11 Other psychoactive substance abuse, in remission.

OUD included: F11.20 Opioid Dependence, uncomplicated; F11.21 Opioid Dependence, in remission.

Co-morbidity was determined based on ICD-10 codes as well as medical history notes (self-report and provider notes). Co-morbidities in this study are non-pregnancy ICD-codes known to affect birth outcomes, complicate pregnancy, or complicate remission/recovery goals.

Physical co-morbidity includes: Hepatitis C, Human Immunodeficiency Virus (HIV), Sexually Transmitted Infections (STI; Chlamydia, genital herpes), hypertension, deep vein thrombosis, anemia, chronic pain,

Psychiatric co-morbidity includes: Depression (any severity), anxiety/generalized anxiety disorder (GAD), post-traumatic stress disorder (PTSD), acute stress disorder, adjustment disorder, bipolar disorder, schizotypal personality disorder, borderline personality disorder, suicidal ideation/suicide attempts.

Substance use history reflects active use at entry into the SUN program or while in the SUN program. Substance use history and tobacco use was abstracted from participant self-report and urine drug screens. Substance use findings were cross referenced with diagnostic codes. Substances were collapsed into three categories based on patterns observed: Opioid use alone, polysubstance use (including opioids; Opioid use was present for 100% of clients), and tobacco. The collapsed categories protect confidentiality (small percentages for some substances). Alcohol use was not reported (yes/no) consistently, alcohol was included in polysubstance use only when diagnosed as alcohol use disorder (ICD-10) or specifically mentioned as dependence or "abuse". Last, a category for disclosed overdose episodes was created based on self-report alone.

Opioid use alone: Pain pills (Vicodin, oxycodone etc.), heroin, tar, fentanyl, buprenorphine prescribed or sourced outside the SUN clinic (incl. Subutex/Suboxone)

Polysubstance use w/ opioids: Opioid(s) from above + one or more of the following: Amphetamine, methamphetamine/crystal meth, cocaine, marijuana, inhalant, LSD, acid, mushrooms, tranquilizer/xylazine, non-prescription benzodiazepines (Xanax, Valium, Klonopin, etc.), non-prescription anticonvulsant (gabapentin).

Tobacco: Cigarettes, vapes, "smoking".

Employment was determined based on participant self-report.

Yes (employment): Full or part time employment at entry into SUN

No (unemployment): No employment at entry into SUN (include clients who are actively seeking employment).

Legal history was determined based on participant self-report. Legal history was collapsed into one category to protect confidentiality. Legal history included self-reported instances of arrest, detention, awaiting court date and/or incarceration.

Trauma history was determined based on participant self-report. Trauma history is shown as a combined category (percentage of participants who reported one or more adverse childhood or traumatic experiences). Trauma history is then broken up into four non-exclusive salient categories (clients may have reported one or more).

Physical abuse: Child maltreatment, child neglect, intimate partner violence, domestic violence/abuse
Sexual abuse: Child sexual abuse, molestation, rape, sexual violence, witnessing sexual violence
Parental substance use: Parental SUD, parental overdose,
Other: Verbal/emotional abuse, family suicide, sibling SUD, foster care

Table 2 - Engagement:

Prior births (parity) is defined as past live births and was determined based on a specific *parity* field in the assessments (when available), notes indicating birth history, (maternal) health history, prior birth outcomes, and/or description of (older) children. Categories were established to ensure sample sizes were large enough to protect confidentiality (0, 1-2, 3+, and missing). The category missing was used when parity could not be established.

Enrollment trimester denotes the gestational trimester of enrollment into the SUN program: First trimester (<13 weeks), second trimester (13-27 weeks, inclusive), third trimester (>27 weeks). Enrollment trimester is established by the date of entry into the program in relationships to the estimated due date or the date of birth and gestational age of the infant.

# of clinical visits describes the total number of visits a participant had to SUN. Number of visits are categorized into four 10-visit increments (1-10; 11-20; 21-30; 31-40), a *still active* category, and *missing* (when number of visits could not be established). This measure is reported as a percentage of participants who fell into each category.

Adherence describes adherence to treatment prescribed by SUN's medical provider and reflects participants active pursuit of recovery. Adherence is established based on the all of the following criteria: Active treatment plan/ active opioid agonist prescription (Subutex or Suboxone), detection of opioid agonist in urine screen, and visit history up till date of delivery. Participants who transferred care to another clinic/facility is considered adhering to treatment.

Table 3 – Social needs:

Social needs (also referred to as Social Determinants of Health (SDOH) by the Sun clinic) is defined as social needs identified by the participant. The categories are created by the SUN clinic and are non-exclusive (a participant can have more than one need). Social needs are shown as *needs identified* and *needs met* for each of the categories.

Transportation, identified: Short or long-term need for reliable transportation.
Transportation, met: SUN clinic provided Uber voucher for visit, SUN partner provided bus pass
Employment, identified: Need for full or part time employment
Employment, met: Referral to a SUN partner

Finance, identified: Short or long-term need for financial support (i.e. rent, car repair)
Finance, met: Referral to a SUN partner (Endless opportunities), rent assistance
Food, identified: Need for nutritional support or to supplement own or family food supply
Food, met: Help with SNAP application, WIC certification
Housing, identified: Need for shelter; need for housing; need for new housing due to safety concerns or unsupportive living situation, co-habitation needs, or need for more space (due to baby).
Housing, met: Referral to a SUN partner (Endless opportunities), referral to in-patient treatment facility, referral to shelter.
Other, identified: In-patient treatment, psychiatric referral, baby items
Other, met: Referral to in-patient treatment, mental health support, or psychiatrist, referral to programs who support new moms.

Table 4 – Birth outcomes:

For birth outcomes, the SUN clinic receives data on all client birth outcomes through care coordination meetings or directly from Atrium health system where the majority clients deliver. In some instances, birth outcomes are reported by the client (self-report). SUN records gestational age in weeks and days and weight in pounds (lbs.) and ounces (oz). Only singleton births were included (n=30).

Preterm denotes preterm birth defined as birth prior to 37 weeks of gestation.

Low birth weight is defined as birth weight less than 5 lbs. 8 oz (2500 grams)
