# Supplementary material for: A credibility-driven evaluation of a community-based perinatal substance use disorder collaborative care model
Source: Front Public Health. 2025 Nov 14;13:1626095. doi: 10.3389/fpubh.2025.1626095 (PMC12660230; doi:10.3389/fpubh.2025.1626095)
Supplement: Supplementary file 3 [file Supplementary_file_3.pdf]

Appendix D: Staff Interview Questions, September 2023

Direct patient care/services

1. “Do you engage with SUN patients differently than other patients?” *(if so, in what ways?)* - “What works well in your interactions and care (*for staff: facilitation)* for SUN patients?”

2. “What are barriers or challenges that you meet when providing care (*alternatively: services*) to SUN patients?”

3. “What are things you have observed (or been told about) that presents a challenge receiving care for SUN patients?”

Organizational support

4. “Thinking about CHA/the SUN clinic (*alternatively: DDS or Atrium)*: What are processes or procedures that facilitate quality patients care/service for SUN patients on a day-to-day basis? - What are processes or procedures that could be improved to facilitate quality patients care for SUN patients overall?” *(prompt: time, staff support, training etc.)*

5. “Do you refer SUN patients to resources or care outside CHA (*alternatively: DDS or Atrium)*?”

b.“What services or care do you typically refer SUN patients to?”

6. “Are there resources or services you wish you could refer to (that you feel is lacking outside CHA/Atrium/DDS)?”

Sustainability (and policy)

7. “Do you think the way you provide care/services for SUN patients/clients today is sustainable over the next 5 years?” *(why/why not)*

8. “If you could change something (anything) to how you provide care/services for SUN patients/clients, what would it be?”

9. “Thinking beyond CHA/SUN clinic (*alternatively: DDS or Atrium)*: Are there changes to structures or policies that you think need to happen in order to provide optimal care/services for pregnant people with SUD?”
