# Supplementary material for: A credibility-driven evaluation of a community-based perinatal substance use disorder collaborative care model
Source: Front Public Health. 2025 Nov 14;13:1626095. doi: 10.3389/fpubh.2025.1626095 (PMC12660230; doi:10.3389/fpubh.2025.1626095)
Supplement: Supplementary file 4 [file Supplementary_file_4.pdf]

Appendix B:

1: SUN participants focus group questions.
2: Survey questions and results by year (2021 and 2023) and combined.

1: SUN participants focus group questions.

Opening: This is a meeting to discuss your experiences seeking treatment with the SUN clinic... Please introduce yourself and tell us how long you have been with SUN/in treatment.

1. Describe what brought you to the SUN clinic, what **led you** to want to seek treatment?
   1A. Discuss stories more in depth.
   1B. How easy or hard was it to make an appointment to see someone?

2. How were you **treated by the doctors** and nurses?
   2A. Do you find that you have to repeat your story or answer the same questions with different people on the treatment staff? If so, does that bother you? How could it be made better?
   2B. Were staff sensitive to your situation?

3. What did you think of your **visits overall?**
   3A. Do you receive a good explanation about what you need to do to manage your health? If not, how could it be improved?

4. What **types of services** do you find to be most helpful to you?
   4A. Who helped you get this service?
   4B. Did many people coordinate this for you?
   4C. Who helped you know what was going to happen next?
   4D. Did you always know who to call for certain services / if you weren't sure about something?
   4E. What other services would you have liked that were not provided or offered?

5. Do you think the quality of care has improved over the course of your treatment?

6. If you wanted to **change anything** about the experience, apart from whether you got better or not, what would you change?

7. Is there any way that **access** to this treatment could be improved?

8. If you're being treated by a team of people within the clinic, do you know everyone's names and roles? Does the team coordinate your care well? If not, how could the coordination be improved?

9.Is there anything we haven't discussed that would **improve your experience** with SUN?

2: SUN participants survey results

Appendix B,2: SUN participants survey results for years 2021-2023						
Q1 (n=29)	Strongly agree	Moderately Agree	Slightly Agree	Slightly Disagree	Strongly Disagree	Missing
I feel **safe** in the SUN Clinic	90% (26)	3% (1)	0%	0%	Co	3% (1)
I was able to begin treatment at the SUN Clinic in a **timely** manner.	90% (26)	3% (1)	0%	0%	3% (1)	3% (1)
I feel like I am receiving the **help and support** I need from SUN Clinic providers	83% (24)	10% (3)	0%	0%	3% (1)	3% (1)
I feel that I can **trust the partners** of the SUN collaboration network	90% (26)	0%	3% (1)	0%	3% (1)	3% (1)
I feel like the people who work here treat me with **respect**	90% (26)	0%	3% (1)	0%	3% (1)	3% (1)
I am **satisfied** with the services that I have received at the SUN Clinic	86% (25)	7% (1)	0%	0%	3% (1)	3% (1)
I feel SUN Clinic providers work well with other agencies to provide me with the **support** that I need	90% (26)	0%	0%	3% (1)	3% (1)	3% (1)
Q2 (n=29)	Extremely Helpful	Moderately Helpful	Slightly Helpful	Moderately Unhelpful	Not helpful/ Not offered	Missing
How helpful is the SUN Project with getting you access to transportation?	72% (21)	7% (2)	3% (1)	0%	10% (3)	7% (2)
How helpful is the SUN Project with getting you access to housing?	59% (17)	3% (1)	7% (2)	0%	18% (5)	14% (4)
How helpful is the SUN Project with getting you access to employment?	55% (16)	3% (1)	7% (2)	3% (1)	21% (6)	14% (4)
How helpful is the SUN Project with getting you access to parenting support?	79% (23)	10% (3)	3% (1)	3% (1)	3% (1)	0%
How helpful is the SUN Project with getting you access to child care assistance?	72% (21)	0%	7% (2)	3% (1)	10% (3)	3% (1)
How helpful is the SUN Project with getting you access to counselling?	93% (27)	3% (1)	0%	3% (1)	0%	0%
How helpful is the SUN with getting you access to relationship/family support?	86% (25)	3% (1)	3% (1)	7% (2)	0%	0%
Q3 (n=29)	Strongly agree	Moderately Agree	Slightly Agree	Slightly Disagree	Strongly Disagree	Missing
**Transportation** problems limit my ability to **access** the SUN Clinic in person.	24% (7)	3% (1)	7% (2)	3% (1)	45% (13)	17% (5)
**Virtual visits** allow me to access services I need at the SUN Clinic I otherwise may not have been able to access	59% (17)	3% (1)	7% (2)	3% (1)	10% (3)	17% (5)
I feel the SUN Clinic has allowed my baby/children to be a part of the treatment and **recovery process**	66% (19)	3% (1)	14% (4)	3% (1)	3% (1)	10% (3)
I feel that the services and support provided by SUN has helped me to be stronger or more **self-reliant**?	76% (22)	10% (3)	0%	0%	3% (1)	10% (3)
***People in my life** limited my ability to access SUN services and support	29% (4)	7% (1)	0%	0%	57% (8)	7% (1)
*I feel the services and support I receive at the SUN clinic will help my long-term **recovery goals**	86% (12)	7% (1)	0%	0%	0%	7% (1)
* Added in 2023						
